# The comparison of the prognostic value of different inflammation-related indicators in patients with oral squamous cell carcinoma

**DOI:** 10.3389/fgene.2025.1652603

**Published:** 2025-08-18

**Authors:** Aoming Cheng, Kaixiong Wei, Qiaoshi Xu, Chong Wang, Bo Li, Huan Liu, Lirui Zhang, Hanchen Zhou, Yu Huang, Yiming Zhang, Xue Zhang, Hao Wang, Chang Liu, Teng Ma, Jingrui Li, Zhengxue Han, Zhien Feng

**Affiliations:** ^1^ Department of Oral and Maxillofacial-Head and Neck Oncology, Beijing Stomatological Hospital, Capital Medical University, Beijing, China; ^2^ Department of Stomatology, Beijing Huairou Hospital, Beijing, China; ^3^ Department of Clinical Laboratory, Beijing Stomatological Hospital, Capital Medical University, Beijing, China

**Keywords:** OSCC, inflammation-related indicators, prognostic nutritional index (PNI), advanced lung cancer inflammation index (ALI), prognosis prediction

## Abstract

**Background:**

This study aimed to determine the prognostic significance of inflammation markers for patients with oral squamous cell carcinoma (OSCC) and sought to analyze the reasons behind it and its value for clinical guidance.

**Methods:**

This study enrolled 624 patients with OSCC who were surgically treated in our hospital. Clinical baseline data and peripheral blood routine/biochemical parameters were collected within 1 week before surgery. The Kaplan-Meier analysis and Cox multifactor regression analysis were performed for statistical analysis. Time-dependent receiver operating characteristic curves (ROC) were used to explore the timeliness of inflammation-related indicators.

**Results:**

The Kaplan-Meier analysis revealed that patients with a high-inflammatory trend had significantly shorter overall survival (OS) and disease-free survival (DFS) compared to the patients with a low-inflammatory trend (*P* < 0.05). Cox multivariate regression analysis demonstrated that advanced lung cancer inflammation index (ALI) and prognostic nutritional index (PNI) were correlated with OS of OSCC patients. Additionally, Subgroup analysis found that ALI and PNI served as independent prognostic factors for OS in male patients. In age-stratified analyses, ALI and PNI also emerged as independent predictors of OS for patients <60 years time-dependent ROC, which ALI demonstrated the best predictive performance for short-term postoperative prognosis (area under the curve (AUC) = 0.74).

**Conclusion:**

ALI and PNI are valuable prognostic indicators in the short term after surgery for young male patients under 60 years old with OSCC.

## 1 Introduction

Oral squamous cell carcinoma (OSCC), the most common head and neck cancer, shows aggressive local invasion and early lymph node metastasis. Despite modern multimodal treatments, 5-year survival rates remain below 60% (Johnson et al., 2020; Ruffin et al., 2023; Bhatia and Burtness, 2023; Jiménez-Labaig et al., 2024; Jiang et al., 2024). This therapeutic impasse underscores an urgent need for the identification of novel, cost-effective, and universally applicable biomarkers that can facilitate early detection, risk stratification, and personalized treatment optimization. The cancer-associated systemic inflammatory response is one of the critical indicators of tumor progression (Bhat et al., 2022). Among the various biomarkers of systemic inflammation, preoperative routine laboratory indicators, including complete blood count and acute-phase reactants, offer several distinct advantages. These parameters are readily available, cost-efficient, highly standardized, and non-invasive, making them ideal candidates for routine clinical use. Previous investigations have extensively explored the prognostic value of serum-based systemic inflammatory markers, demonstrating their potential in predicting patient outcomes across multiple cancer types (Dupré et al., 2018; Tampa et al., 2018; Wu et al., 2022; Battista et al., 2024; Yang et al., 2019; Feng et al., 2024). The development of an optimal biomarker useful for the prediction of recurrence or poor prognosis is clinically important to identify patients at potential risk. Notably, however, the potential of preoperative routine hematological parameters to accurately predict the prognosis and clinical outcomes of OSCC patients is still not fully understood. Due to the significant variability in OSCC, it is crucial to systematically assess the predictive value of these accessible biomarkers, which can help identify high-risk patients early and facilitate personalized treatment approaches.

Today, the relationship between inflammation, innate immunity, and cancer is more widely accepted. The inflammatory response is closely linked to the immune status. In tumors, chronic and persistent inflammation often weakens the body’s innate immune system. At the same time, it enhances tumor-mediated immunity, aiding tumor growth, local invasion, new blood vessel formation, and distant metastasis (Cao and Kagan, 2023). Numerous studies have shown that strong inflammatory processes significantly influence tumor progression. Correspondingly, the state of systemic inflammatory burden may also reflect the degree of tumor progression and prognosis. Previous studies have indicated that the indicators related to preoperative routine hematological parameters, such as Advanced lung cancer inflammation index, prognostic nutritional index and et al., which are readily derivable through routine hematological assessments in cancer patients, are effective prognostic markers (Wei et al., 2021; Lin et al., 2022; Gaudioso et al., 2021; Murad et al., 2022; Valero et al., 2020). Therefore, investigating the correlations between inflammation-related biomarkers and the prognosis of OSCC, as well as deciphering the potential connection between high-risk patient profiles and persistent inflammatory states, represents a highly promising and clinically relevant avenue with broad prospects for clinical application.

Herein, this study conducted a single-institution retrospective cohort study, comprising 624 primary OSCC patients who underwent treatment at Beijing Stomatological Hospital between 2016 and 2021. Clinical baseline data and peripheral blood routine/biochemical parameters collected within 1 week before surgery were retrospectively retrieved. Preoperative inflammation-related indicators, including neutrophil-lymphocyte ratio (NLR), lymphocyte-monocyte ratio (LMR), systemic inflammation score (SIS), systemic immune-inflammation index (SII), platelet-lymphocyte ratio (PLR), Advanced lung cancer inflammation index (ALI), prognostic nutritional index (PNI), and H-index, were calculated. Patients underwent systematic follow-up, with prognostic values assessed via Kaplan-Meier survival analysis and multivariate Cox proportional hazards regression models. The analysis showed that ALI and PNI could serve as independent prognostic factors for OSCC patients, especially demonstrating stronger predictive efficacy in short-term postoperative outcomes and male patients under 60 years.

## 2 Patients and methods

### 2.1 Patients

**FIGURE 1:**
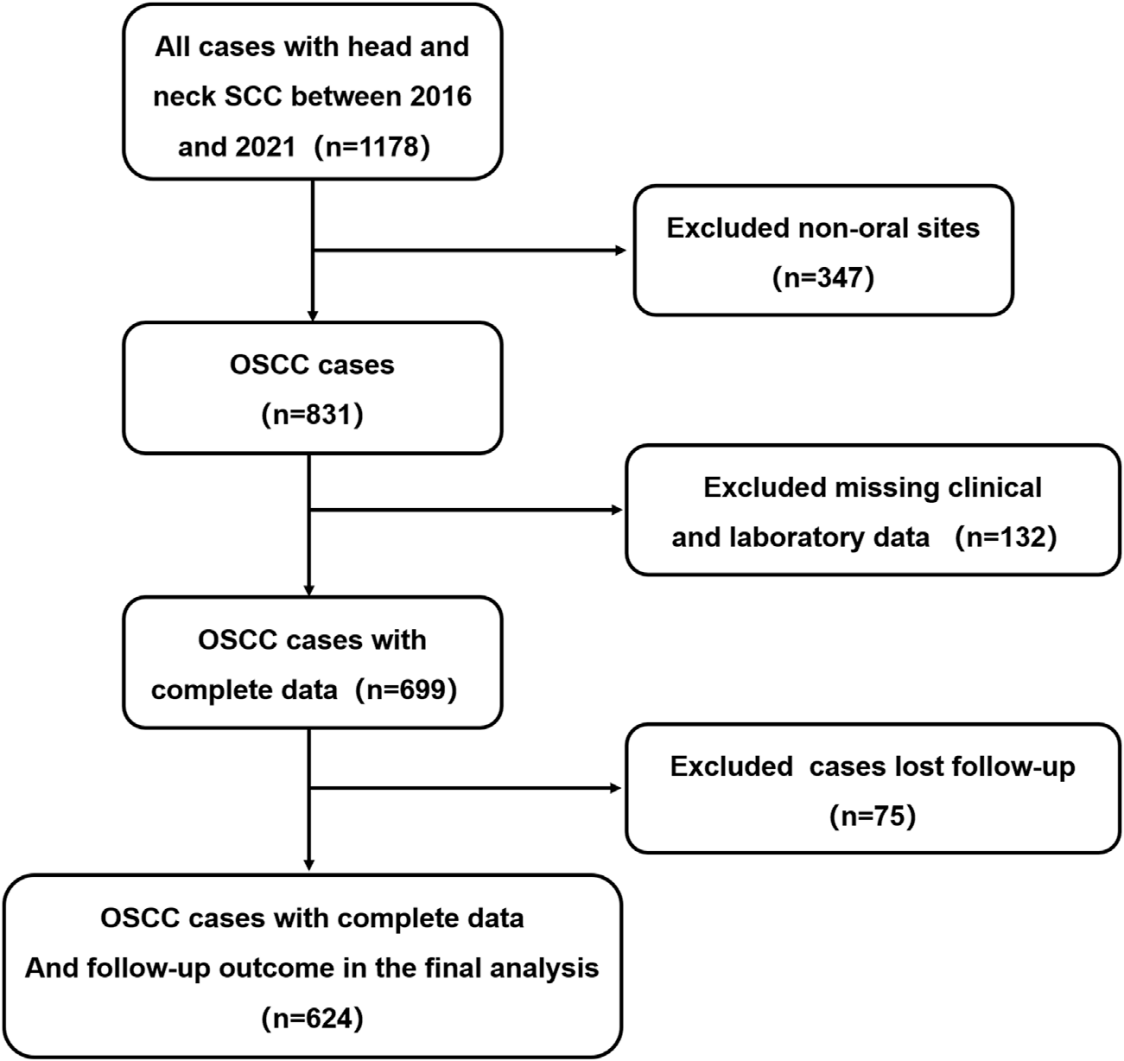
Flowchart of 624 patients enrolled in this study.

Our study was conducted in full accordance with ethical principles, including the World Medical Association’s Declaration of Helsinki (2002 version), with IRB approval (CMUSH-IRB-KJ-PJ-2024-29). We performed a single-institution, retrospective cohort study. From January 2016 to December 2021, patients treated in Beijing Stomatological Hospital who satisfied the following inclusion criteria were included. The process for selecting patients for model development is presented in Figure 1. The inclusion criteria were as follows: 1) patients pathologically diagnosed as squamous cell carcinoma. 2) With a tumor located on the tongue, lower gingiva, upper gingiva, buccal mucosa, floor of the mouth, or hard palate. 3) No preoperative chemotherapy, radiotherapy or other radical treatments before the operation; Relevant routine laboratory indicators of peripheral blood should be examined within 7 days before the operation; Patients were excluded if the follow-up time was less than 1 year, and complete clinical and laboratory data were not completed.

### 2.2 Data collection

The dataset includes patients’ baseline information (age, gender, 8^th^ AJCC TNM stage, tobacco and alcohol use), Preoperative routine hematological parameters within 7 days (absolute neutrophil count, absolute monocyte count, absolute lymphocyte count, platelet count, albumin, hemoglobin). and follow-up data.

### 2.3 Evaluation for indicators related to preoperative routine hematological parameters

All the indicators mentioned in our study were evaluated and collected as follows:

(1) Neutrophil-to-Lymphocyte Ratio (NLR) = Absolute neutrophil count/Absolute lymphocyte count
(2) Platelet-to-Lymphocyte Ratio (PLR) = Platelet count/Absolute lymphocyte count
(3) Lymphocyte-to-Monocyte Ratio (LMR) = Absolute lymphocyte count/Absolute monocyte count
(4) Systemic Immune-Inflammation Index (SII) = (Platelet count × Absolute neutrophil count)/Absolute lymphocyte count
(5) Prognostic Nutritional Index (PNI) = Albumin +5 × Absolute lymphocyte count
(6) Advanced Lung Cancer Inflammation Index (ALI) = [Body Mass Index (BMI) × Albumin]/NLR
(7) Systemic Inflammation Marker (SIM)/Systemic Inflammation Response Index (SIRI) = (Absolute neutrophil count × Absolute monocyte count)/Absolute lymphocyte count
(8) H-index = [Absolute neutrophil count × Absolute monocyte count]/[Absolute lymphocyte count × Hemoglobin × Albumin] × 10^4^.

### 2.4 Follow-up

Patients were followed for at least 1 year after the operation, and phone call records or visits to the clinic were used for the verification of disease and survival status.

### 2.5 Statistical analysis

Normally distributed continuous data are reported as the means ± standard deviation (SD). Qualitative data are described by frequency and percentage. the outcome assessment parameters included overall survival (OS) and disease-free survival (DFS). DFS was defined as the time from the first day after treatment to the time of disease progression (including local/regional recurrence, second primary or distant metastasis) or death due to any cause. OS was defined as the period from the end of treatment until death caused by any reason. A receiver operating characteristic (ROC) curve was used to determine the optimal cutoff values for the indicators related to preoperative routine hematological parameters. The Kaplan-Meier method was used to compare the outcomes of the different risk groups Statistical significance was determined with the log-rank test, to explore the timeliness of each indicator, time-dependent receiver operating characteristic curves were constructed using the “survival ROC” package in R software, with calculation of the area under the curve (AUC) for postoperative survival at 1–5 years. The relevant forest plots were drawn using the “forest plot” package in R software. The analyses were performed with SPSS 22.0 (IBM, Chicago, IL, United States), and a two-sided *P*-value <0.05 was considered significant.

## 3 Results

### 3.1 Patient characteristics

According to the inclusion criteria, we identified a total of 624 patients with OSCC who underwent primary surgery with surgically negative margins. Among these, 354 patients were men and 270 were women, 1:1.31. The most common tumor site was the tongue (39.7%), followed by the gingiva (26.4%). The proportions of smokers and drinkers were 41.7% and 34.5% respectively. From the clinical staging perspective, the cohort included 59 cases of Tis (9.5%), 98 Stage I cases (15.7%), 150 Stage II cases (24.0%), 68 Stage III cases (10.9%), and 249 Stage IV cases (39.9%), while the pathological T staging comprised 62 Tis cases (9.9%), 87 T1 cases (13.9%), 174 T2 cases (27.9%), 94 T3 cases (15.1%), and 207 T4 cases (33.2%).

For the indicators related to preoperative routine hematological parameters, the mean NLR was 2.09 ± 0.06, and for the other indicators, the data were as follows: mean PLR: 131.85 ± 2.03, mean LMR:4.52 ± 0.07, mean SII: 470.0 ± 14.74, mean PNI: 45.61 ± 0.18, mean ALI:54.60 ± 1.13, mean SIM:0.95 ± 0.06, and H-index: 2.19 ± 0.29. Further, the mentioned continuous variables were converted to binary classifications by cutoff values as follows: NLR (2.3), PLR (132.7), LMR (3.0), SII (661.6), PNI (47.3), ALI (40.0), SIM (1.6), and H-index (2.7). Previous studies have demonstrated that inflammatory states are characterized by elevated absolute neutrophil count, absolute monocyte count, and platelet count, coupled with reduced serum albumin and absolute lymphocyte count (Wu et al., 2022; Yang et al., 2019; Sakai et al., 2023; Cao and Kagan, 2023; Wei et al., 2021; Lin et al., 2022; Gaudioso et al., 2021; Murad et al., 2022; Valero et al., 2020; Diao et al., 2018; Park et al., 2018). Consequently, based on established formulas for these parameters, patients with elevated NLR, PLR, SII, SIM, and H-index, or reduced LMR, PNI, and ALI, were categorized as having a high-inflammatory trend. The remaining cases were classified as having a low-inflammatory trend.

Through February 2023, the end of the follow-up, the median follow-up time was 35months (IQR: 23-52). During the follow-up period, a total of 116 death events were observed (all-cause mortality rate 18.6%), of which 199 patients (31.9%) developed local recurrence or lymph node metastasis. The baseline characteristics of the patients are summarized in Table 1.

**TABLE 1:** Patient baseline data characteristics.

Variable	n	%
	624	100
Gender		
Male	354	56.7
Female	270	43.3
Age		
≥60	367	58.8
<60	257	41.2
Sites		
Tongue	248	39.7
Gingiva	165	26.4
Buccal	96	15.5
Others	115	18.4
Growth pattern		
Exogenous	190	30.4
ulcerative	231	37.1
Infiltrating type	203	32.5
pT(AJCC 8th)		
Tis	62	9.9
1	87	13.9
2	174	27.9
3	94	15.1
4	207	33.2
pN (AJCC 8th)		
0	443	71.0
1	73	11.7
2	80	12.8
3	28	4.5
Clinical stages		
0	59	9.5
I	98	15.7
II	150	24.0
III	68	10.9
IV	249	39.9
Drinking history		
Drinker	215	34.5
Non-drinker	409	65.5
Smoking history		
Smoker	260	41.7
Non-smoker	364	58.3
NLR: mean ± SD	2.09 ± 0.06	
PLR: mean ± SD	131.85 ± 2.03	
SII: mean ± SD	470.0 ± 14.74	
PNI: mean ± SD	45.61 ± 0.18	
ALI: mean ± SD	54.60 ± 1.13	
SIM: mean ± SD	0.95 ± 0.06	
H-index mean ± SD	2.19 ± 0.29	
LMR: mean ± SD	4.52 ± 0.07	

### 3.2 Significance of inflammation-related indicators for OS and DFS

**FIGURE 2:**
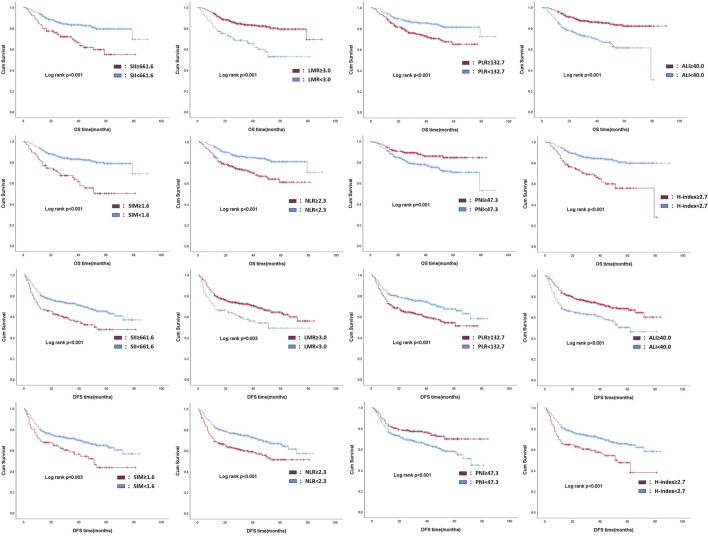
Kaplan-Meier analysis of prognostic outcome in patients with different inflammatory trends, according to each inflammation-related indicator.

To evaluate the impact of preoperative inflammatory status on clinical outcomes, we performed Kaplan-Meier survival analysis comparing postoperative OS and DFS between patients with high- and low-inflammatory trend as mentioned above. The survival curves showed significantly worse OS and DFS in patients with high-inflammatory trends in each inflammatory-related indicator (log-rank test, *P* < 0.005 for both endpoints), as illustrated in Figure 2. This result suggests that the preoperative systemic inflammatory status may be an independent risk factor affecting the survival prognosis of patients with OSCC.

Supported by the above evidence, we further analyzed the prognostic predictive ability of each indicator item by item based on Cox univariate or multivariate analysis. Before applying the COX univariate and multivariate regression analysis, the proportional hazards of each index were verified (Supplementary Figure S1). Univariate Cox regression analysis of postoperative overall survival (OS) identified significant associations with the following variables: clinical stage (III vs 0, HR = 8.523, 95% CI: 1.080–67.277,*P* = 0.042), and inflammation-related indicators including NLR (high vs. low: HR = 2.599, 95% CI: 1.776-3.686, *P* < 0.001), PLR (high vs. low: HR = 1.986, 95% CI: 1.374-2.869, *P* < 0.001), LMR (high vs. low: HR = 0.345, 95% CI: 0.232-0.513, *P* < 0.001), SII (high vs. low: HR = 2.593, 95% CI: 1.759-3.822, *P* < 0.001), PNI (high vs. low: HR = 0.388, 95% CI: 0.242-0.623, *P* < 0.001), ALI (high vs. low: HR = 0.300, 95% CI: 0.207–0.434, *P* < 0.001), SIM (high vs. low: HR = 3.083, 95% CI: 2.034–4.672, *P* < 0.001), and H-index (high vs low: HR = 3.275, 95% CI: 2.246–4.776, *P* < 0.001). After adjusting for confounding factors, we performed Multivariate Cox regression analysis and confirmed that 2 inflammation-related indicators served as independent prognostic factors for OS in OSCC patients (Table 2). Specifically, elevated levels of PNI (high vs low: HR = 0.583, 95% CI: 0.348–0.977, *P* = 0.041), and ALI (high vs low: HR = 0.502, 95% CI: 0.282–0.892, *P* = 0.019) demonstrated significant correlations with OS, indicating that elevated levels of these markers may correlate with patient survival (Table 2).

**TABLE 2:** Univariate and multivariate COX regression analysis of risk factors for postoperative OS in OSCC patients.

Variable	Univariate analysis		Multivariate analysis	
	HR (95%CI)	*P*-value	HR (95%CI)	*P*-value
Gender				
male vs female	1.225(0.843–1.781)	0.288		
Age				
≥60 vs<60	1.435 (0.977–2.106)	0.065		
Smoking history				
Smoker vs non-smoker	1.127 (0.781–1.626)	0.522		
Drinking history				
Drinker vs non-drinker	1.101 (0.755–1.606)	0.618		
Sites		0.165		
Tongue	Ref.			
Gingiva	1.494 (0.966–2.309)	0.071		
Buccal	1.137 (0.653–1.981)	0.650		
Others	0.841 (0.478–1.482)	0.550		
Growth pattern		0.331		
Exogenous	Ref.			
ulcerative	1.199 (0.747–1.925)	0.453		
Infiltrating type	1.422 (0.891–2.268)	0.140		
Clinical stages		<0.001		<0.001
0	Ref.		Ref	
I	4.398 (0.541–35.750)	0.166	4.216(0.516–34.460)	0.180
II	5.942 (0.781–45.19)	0.085	6.005(0.787–45 813)	0.084
III	8.523 (1.080–67.277)	0.042	7.747(0.977–61.413)	0.054
IV	24.598 (3.425–176.663)	0.001	18.811(2.590–136.608)	0.004
NLR				
High vs Low	2.599 (1.776–3.686)	<0.001	0.920(0.504–1.683)	0.788
PLR				
High vs Low	1.986 (1.374–2.869)	<0.001	0.993(0.635–1.554)	0.976
LMR				
High vs Low	0.345 (0.232–0.513)	<0.001	1.171(0.602–2.280)	0.641
SII				
High vs Low	2.593 (1.759–3.822)	<0.001	0.947(0.535–1.677)	0.852
PNI				
High vs Low	0.388 (0.242–0.623)	<0.001	0.583(0.348–0.977)	0.041
ALI				
High vs Low	0.300 (0.207–0.434)	<0.001	0.502(0.282–0.892)	0.019
SIM				
High vs Low	3.083 (2.034–4.672)	<0.001	1.575(0.710–3.492)	0.264
H-index				
High vs Low	3.275 (2.246–4.776)	<0.001	1.104(0.539–2.258)	0.787

**FIGURE 3:**
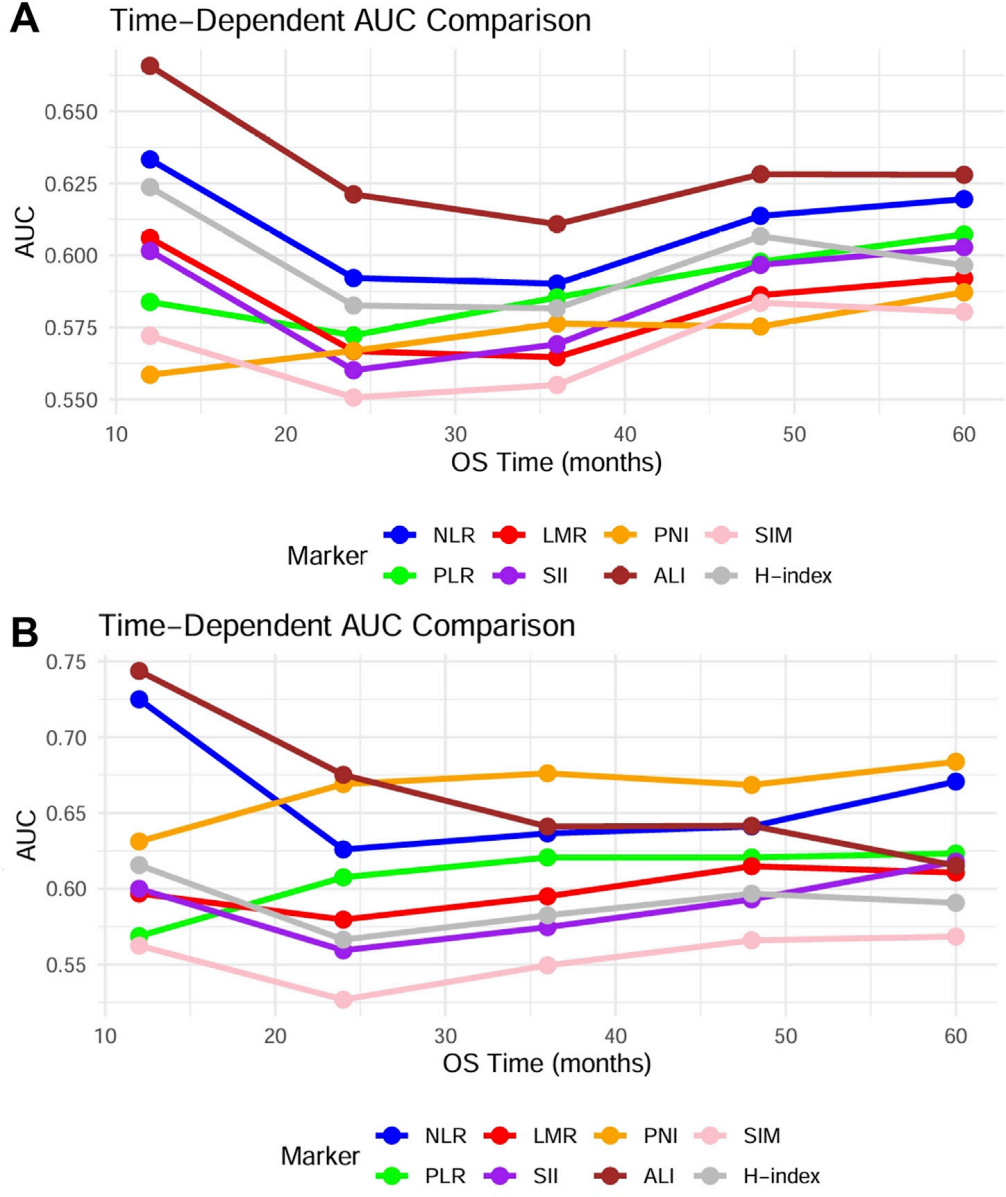
Using time-dependent ROC analysis, the timeliness of the mentioned indicators is assessed. **(A)** Time-dependent ROC of 8 indicators in 624 patients. **(B)** Time-dependent ROC of 8 indicators in patients under 60 years and the younger male subgroups.

Similarly, Univariate Cox regression analysis of postoperative DFS risk factors demonstrated significant associations with sex, age, smoking and drinking history, clinical stage, and all inflammation-related markers (Supplementary Table S1). Multivariate Cox regression analysis after adjusting for confounding factors confirmed that only clinical stage served as an independent prognostic factor for DFS in OSCC patients (Supplementary Table S1). This result indicated that all inflammation-related indicators only influence the overall survival status of patients. In addition, time-dependent ROC revealed that among the eight included indicators, the ALI demonstrated the optimal predictive performance, achieving a 1-year postoperative AUC of 0.65, though no statistically significant differences were observed between the indicator groups. Notably, seven preoperative markers—NLR, LMR, SII, ALI, PLR, SIM, and H-index—exhibited a significant decline in AUC values at the 2-year postoperative interval compared to the 1-year results (Figure 3A), suggesting that these preoperative indicators may only hold predictive value for short-term prognosis after treatment.

### 3.3 Evaluation of prognostic significance of inflammation-related indicators in age-specific and gender-specific subgroups

In order to further clarify the prognostic value of the above indicators and minimize the influence of potential confounding factors, we conducted stratified subgroup analyses using multivariate Cox proportional hazards models to evaluate age-specific (<60 vs. ≥60 years) and gender-specific prognostic associations. The indicators related to preoperative routine hematological parameters demonstrated differential predictive efficacy across subgroups, with PNI (HR = 0.256, 95% CI = 0.125–0.522, *P* < 0.001), ALI (HR = 0.372, 95% CI = 0.211–0.658, *P* = 0.001), along with clinical stage showing independent associations with postoperative OS in younger patients (<60 years, n = 257). Similarly, PNI also correlated with DFS in younger patients. In contrast, none exhibited significant prognostic value for OS or DFS in elderly patients (≥60 years, n = 367), indicating age-dependent prognostic relevance of preoperative inflammation-related indicators in OSCC (Table 3; Supplementary Tables S2-S4).

**TABLE 3:** Univariate and multivariate COX regression analysis of risk factors for postoperative OS in OSCC younger patients (<60 years**)**.

Variable	Univariate analysis		Multivariate analysis	
	HR (95%CI)	*P*-value	HR (95%CI)	*P*-value
Gender				
male vs female	3.478(1.461–8.278)	0.005	2.472(0.867–7.045)	0.090
Smoking history				
Smoker vs non-smoker	1.740(0.928–3.264)	0.084		
Drinking history				
Drinker vs non-drinker	2.077(1.118–3.859)	0.021	0.944(0.451–1.979)	0.88
Sites		0.967		
Tongue	Ref.			
Gingiva	1.217 (0.532–2.783)	0.641		
Buccal	1.097 (0.404–2.973)	0.856		
Others	1.152 (0.520–2.553)	0.727		
Growth pattern		0.642		
Exogenous	Ref.			
ulcerative	0.961 (0.420–2.203)	0.926		
Infiltrating type	1.316 (0.606–2.860)	0.488		
Clinical stages		<0.001		0.045
0	Ref.		Ref.	
I	3410.085(0.001-4.29E+53)	0.890	7944.894(0.001-3019E+68)	0.906
II	4023.983(0.001-5.04E+53)	0.888	11777.170(0.001-4.464E+68)	0.902
III	14560.021(0.001-1.824E+54)	0.871	25459.404(0.001-9.634E+68)	0.894
IV	23570.520(0.001-3.170E+54)	0.863	46166.396(0.001-1.746E+69)	0.887
NLR				
High vs Low	4.216 (2.264–7.851)	<0.001	1.498 (0.570–3.939)	0.413
PLR				
High vs Low	1.822 (0.979–3.392)	0.058		
LMR				
High vs Low	0.203 (0.106–0.390)	<0.001	1.150(0.400–3.304)	0.795
SII				
High vs Low	2.818 (1.406–5.648)	0.003	0.969(0.312–3.010)	0.956
PNI				
High vs Low	0.123 (0.044–0.347)	<0.001	0.119(0.039–0.371)	<0.001
ALI				
High vs Low	0.157 (0.083–0.297)	<0.001	0.342(0.136–0.856)	0.022
SIM				
High vs Low	3.785 (1.850–7.746)	<0.001	2.218(0.516–9.535)	0.285
H-index				
High vs Low	4.495 (2.378–8.495)	<0.001	0.524(0.159–1.728)	0.288

Similarly, in the male subgroup (n = 354), multivariate-adjusted analysis demonstrated that 2 indicators independently influenced postoperative OS, including PNI: HR = 0.371, 95% CI = 0.175- 0.789, *P* = 0.010, ALI: HR = 0.468, 95% CI = 0.223–0.980, *P* = 0.044. However, only the clinical stages serve as independent prognostic factors for DFS. Conversely, in female patients (n = 270), none of these biomarkers showed independent prognostic significance for either OS or DFS (*P* > 0.05) (Table 4; Supplementary Tables S5-S7).

**TABLE 4:** Univariate and multivariate COX regression analysis of risk factors for postoperative OS in male patients.

Variable	Univariate analysis		Multivariate analysis	
	HR (95%CI)	*P*-value	HR (95%CI)	*P*-value
Age				
<60 years vs ≥60 years	0.998(0.626–1.593)	0.995		
Smoking history				
Smoker vs non-smoker	0.916(0.554–1.516)	0.734		
Drinking history				
Drinker vs non-drinker	1.051(0.653–1.691)	0.839		
Sites		0.479		
Tongue	Ref.			
Gingiva	1.125(0.622–2.032)	0.697		
Buccal	0.950 (0.468–1.929)	0.888		
Others	0.676 (0.358–1.277)	0.228		
Growth pattern		0.215		
Exogenous	Ref.			
ulcerative	1.526 (0.803–2.900)	0.197		
Infiltrating type	1.723 (0.910–3.265)	0.095		
Clinical stages		<0.001		0.150
0	Ref.		Ref.	
I	12106.543(0.001-8.866E+57)	0.882	12106.543 (0.001-2.234E+63)	0.889
II	13974.963(0.001-1.021E+58)	0.880	17067.148 (0.001-2.234E+63)	0.883
III	27605.229(0.001-2.017E+58)	0.872	27219.716 (0.001-2.234E+63)	0.880
IV	44940.295(0.001-3.280E+58)	0.866	36182.389 (0.001-2.234E+63)	0.887
NLR				
High vs Low	3.140 (1.950–5.057)	<0.001	1.151 (0.545–2.428)	0.713
PLR				
High vs Low	2.352 (1.461–3.787)	<0.001	0.876 (0.483–1.588)	0.662
LMR				
High vs Low	0.283 (0.177–0.455)	<0.001	0.768 (0.348–1.696)	0.514
SII				
High vs Low	2.861 (1.764–4.642)	<0.001	1.011 (0.478–2.139)	0.976
PNI				
High vs Low	0.227 (0.113–0.458)	<0.001	0.371 (0.175–0.789)	0.010
ALI				
High vs Low	0.240 (0.145–0.398)	<0.001	0.468 (0.223–0.980)	0.044
SIM				
High vs Low	3.148 (1.912–5.183)	<0.001	1.166 (0.446–3.046)	0.754
H-index				
High vs Low	3.466 (2.160–5.563)	<0.001	0.962 (0.385–2.406)	0.934

**FIGURE 4:**
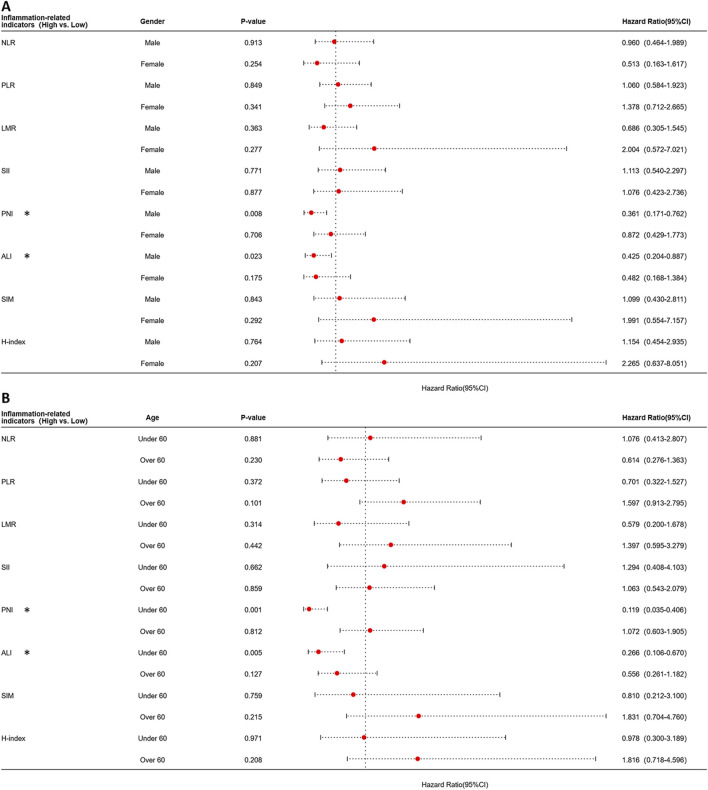
Forest plot of subgroup analysis stratified by age and gender for each indicator using the Cox proportional hazards model. **(A)** Forest plot stratified by gender. **(B)** Forest plot stratified by age.

Building upon these findings, the aforementioned indicators demonstrated significant prognostic predictive value exclusively in patients under 60 years and the younger male subgroups. To evaluate specific populations, we further constructed time-dependent ROC curves and systematically calculated AUC values at critical postoperative intervals (1-year, 3-year, and 5-year). Analytical results revealed that compared to the overall population, all indicators exhibited enhanced predictive efficacy in this targeted subgroup despite maintaining analogous prognostic trends. Notably, ALI demonstrated superior predictive performance among all parameters, achieving an AUC of 0.74 during the first postoperative year, significantly outperforming other indicators (Figure 3B). Furthermore, we evaluated the differences in prognostic effects of the 8 indicators among patients of different ages and genders through interaction analysis. Based on the Cox proportional hazards regression model, patients were divided into different subgroups by age (≥60 years vs. <60 years) and gender. The HR and 95%CI of each indicator in each subgroup were calculated, and the interaction P-values were used to assess the statistical differences of the 8 indicators across different stages and subgroups. Forest plots visualizing the results indicated that ALI and PNI, like previously mentioned, also demonstrated statistically significant effects in patients under 60 years old and in male patients (Figure 4). This discovery suggests ALI and PNI may serve as clinically valuable biomarkers for prognostic evaluation in male OSCC patients under 60 years.

## 4 Discussion

Globally, the incidence of OSCC has consistently increased, with its 5-year survival rate stagnating between 50% and 60%. Against this backdrop, identifying economical, effective, and highly accessible prognostic prediction indicators allows clinicians to more accurately and quickly assess patient outcomes. This also provides a crucial foundation for personalized treatment plans for OSCC patients. Additionally, previous studies have confirmed that increased infiltration of local tumor immune cells, combined with high systemic inflammatory burden levels, serves as a significant prognostic indicator for cancer progression (Diao et al., 2018). Taken together, preoperative inflammation-related indicators—as economical, easily obtainable, non-invasive, and safe indicators—can effectively guide clinicians in formulating preoperative intervention plans for OSCC patients. This holds substantial value for reducing the risk of perioperative complications and improving long-term prognosis in OSCC patients. In recent years, the role of preoperative inflammation-related indicators in predicting tumor prognosis has garnered increasing attention. Previous studies have demonstrated that the PLR can serve as an independent prognostic factor in OSCC patients (Park et al., 2018), while the PNI, SIM, and ALI have shown specific predictive value in the prognosis of human papillomavirus (HPV)-negative OSCC patients (Ruiz-Ranz et al., 2022). Battista et al. found that the NLR is more accurate than the LMR and PLR in predicting OSCC prognosis, with further research confirming that LMR significantly outperforms traditional biomarkers such as albumin and C-reactive protein in predicting OSCC outcomes (Battista et al., 2024). However, existing studies have certain limitations: most feature small sample sizes (fewer than 300 cases), limited markers included, and a lack of distinction or comparison of predictive capabilities across different OSCC patient subgroups or time points, leading to significant discrepancies in research conclusions.

Our retrospective study analyzed 624 OSCC patients treated at our hospital between 2016 and 2021, collecting baseline data and preoperative peripheral blood test indices. Through systematic evaluation, we thoroughly analyzed the associations between preoperative inflammation-related markers and long-term prognostic outcomes in OSCC patients, leveraging the study’s strengths of a long temporal span and large sample size. A total of 8 inflammation-related indicators were included. By constructing subgroup analysis models stratified by demographic characteristics (age/gender), we systematically evaluated the population heterogeneity in the predictive efficacy of each marker, screening for predictive factors with optimal discriminative power, and identifying their sensitive populations. This provides an evidence-based foundation for developing targeted perioperative inflammatory intervention plans to achieve personalized prognostic stratification management across different patient subgroups.

More precisely, our study uncovered significant age- and gender-specific heterogeneity in the prognostic utility of preoperative inflammation-related indicators through stratified analyses. In age-stratified subgroups, PNI (HR = 0.256, 95% CI = 0.125–0.522, p < 0.001) and ALI (HR = 0.372, 95% CI = 0.211–0.658, p = 0.001) demonstrated robust predictive value for post-operative OS in OSCC patients under 60 years, whereas no significant associations were observed in those aged ≥60 years (p > 0.05). This age-dependent discrepancy is likely rooted in immunosenescence and chronic low-grade inflammation inherent to aging populations. Older adults exhibit persistent elevation of proinflammatory cytokines such as IL-6 and TNF-α, and impaired inflammation resolution mechanisms, which creates a pathological microenvironment that masks tumor-specific inflammatory signals (Gaudioso et al., 2021; Valero et al., 2020; Fang et al., 2022; Wu et al., 2020; Wang et al., 2021). Concurrently, aging-related metabolic dysregulations—such as visceral adiposity, insulin resistance, and declining sex hormones—exacerbate baseline inflammatory burden, diluting the discriminative power of these markers for oncological outcomes (Boscolo-Rizzo et al., 2021; Adnan et al., 2022). In contrast, younger patients maintain dynamic cytokine homeostasis and self-limiting immune responses, enabling inflammation markers to more accurately reflect tumor biology and host immune-tumor interactions (Gaudioso et al., 2021). Gender-stratified analysis further revealed that PNI (HR = 0.371, 95% CI = 0.175- 0.789, p = 0.010) and ALI (HR = 0.468, 95% CI = 0.223–0.980, p = 0.044) also served as independent predictors of OS exclusively in male patients, with no prognostic significance detected in females (p > 0.05). This divergence may be related and aligns with hormonal immunomodulation. Previous studies indicated that androgens suppress inflammation by regulating immune cell function, which is negatively correlated with systemic inflammation indices, while estrogen promotes hepatic synthesis of proinflammatory mediators such as IL-6 and C-reactive protein (Mirili et al., 2019; Al-Jawadi et al., 2018). These findings concur with the threefold higher prevalence of autoimmune diseases in females (Al-Jawadi et al., 2018), reflecting fundamental gender differences in inflammatory reaction. Additionally, sex hormone–mediated regulation of the hypothalamic-pituitary-adrenal axis and glucocorticoid receptor signaling may amplify stress-induced inflammatory responses in males during tumorigenesis (Mirili et al., 2019), as exemplified by the superior diagnostic value of combined NLR/PLR in male early gastric cancer patients (Ahn et al., 2019). Collectively, these observations underscore the critical role of biological sex in shaping inflammatory responses to neoplasia. Notably, our study has certain limitations. Preoperative markers exhibited stronger predictive efficacy for OSCC outcomes in the short term (≤1 year), with their utility diminishing thereafter. This phenomenon may stem from their static nature: these indicators merely capture the baseline inflammatory and nutritional status, failing to reflect postoperative metabolic changes, chronic inflammation, or the impacts of therapeutic interventions such as chemotherapy and radiotherapy. Additionally, in retrospective study designs, uncontrollable factors such as information bias introduce instability, further restricting the long-term validity of these markers. Despite these constraints, combining markers with clinicopathologic data may enhance short-term risk stratification, enabling personalized perioperative inflammation management to improve long-term prognostic outcomes.

## 5 Conclusion

The results of this study suggest that ALI and PNI are valuable prognostic indicators in the short term after surgery for OSCC patients, particularly among young males under 60 years old. Based on these findings, in younger male patients with OSCC, we recommend closer monitoring of preoperative inflammatory indicators and implementation of targeted preoperative interventions to modulate inflammatory status, which may potentially enhance postoperative outcomes.

## Data Availability

The data that supports the findings of this study are available upon reasonable request. Requests to access the datasets should be directed to the corresponding author.
