# Supplementary material for: The comparison of the prognostic value of different inflammation-related indicators in patients with oral squamous cell carcinoma
Source: Front Genet. 2025 Aug 18;16:1652603. doi: 10.3389/fgene.2025.1652603 (PMC12399640; doi:10.3389/fgene.2025.1652603)
Supplement: Supplementary file 1 [file Table1.docx]

**Supplementary table 1: Univariate and multivariate COX regression analysis of risk factors for postoperative DFS in OSCC patients**

|  | **Univariate analysis** | |  | **Multivariate analysis** | |
| --- | --- | --- | --- | --- | --- |
|  | **HR（95%CI）** | ***P*-value** |  | **HR（95%CI）** | ***P*-value** |
| **Gender**  **male vs. female** | **0.954（0.721-1.262）** | **0.740** |  |  |  |
| **Age**  **≥60 vs.<60** | **1.144（0.861-1.521）** | **0.353** |  |  |  |
| **Smoking history**  **Smoker vs. non-smoker** | **0.920（0.694-1.222）** | **0.565** |  |  |  |
| **Drinking history**  **Drinker vs. non-drinker** | **1.074（0.805-1.433）** | **0.628** |  |  |  |
| **Sites** |  | **0.196** |  |  |  |
| **Tongue** | **Ref.** |  |  |  |  |
| **Gingiva** | **1.402 (0.997-1.972)** | **0.052** |  |  |  |
| **Buccal** | **1.230 (0.810-1.868)** | **0.331** |  |  |  |
| **Others** | **0.985 (0.655-1.483)** | **0.944** |  |  |  |
| **Growth pattern** |  | **0.283** |  |  |  |
| **Exogenous** | **Ref.** |  |  |  |  |
| **ulcerative** | **0.881 (0.619-1.254)** | **0.482** |  |  |  |
| **Infiltrating type** | **1.152 (0.818-1.623)** | **0.419** |  |  |  |
| **Clinical stages** |  | **<0.001** |  |  | **<0.001** |
| **0** | **Ref.** |  |  | **Ref.** |  |
| **I** | **1.829 (0.857-3.904)** | **0.119** |  | **1.781(0.831-3.819)** | **0.138** |
| **II** | **1.559 (0.746-3.259)** | **0.238** |  | **1.576(0.751-3.306)** | **0.229** |
| **III** | **1.634 (0.722-3.698)** | **0.239** |  | **1.604(0.705-3.649)** | **0.259** |
| **IV** | **3.716 (1.885-7.325)** | **<0.001** |  | **3.105(1.556-6.194)** | **0.001** |
| **NLR**  **High vs. Low** | **1.822 (1.373-2.417)** | **<0.001** |  | **1.008 (0.628-1.618)** | **0.973** |
| **PLR**  **High vs. Low** | **1.589 (1.203-2.099)** | **0.001** |  | **1.096 (0.785-1.530)** | **0.590** |
| **LMR**  **High vs. Low** | **0.581 (0.412-0.819)** | **0.002** |  | **1.244（0.720-2.149）** | **0.434** |
| **SII**  **High vs. Low** | **1.868 (1.355-2.576)** | **<0.001** |  | **1.019（0.641-1.618）** | **0.937** |
| **PNI**  **High vs. Low** | **0.568 (0.411-0.784)** | **0.001** |  | **0.603（0.395-0.920）** | **0.065** |
| **ALI**  **High vs. Low** | **0.486 (0.368-0.643)** | **<0.001** |  | **0.707（0.504-1.020）** | **0.138** |
| **SIM**  **High vs. Low** | **1.786 (1.234-2.584)** | **0.001** |  | **1.002（0.518-1.936）** | **0.995** |
| **H-index**  **High vs. Low** | **2.100 (1.532-2.877)** | **<0.001** |  | **1.343（0.748-2.411）** | **0.324** |

**Supplementary table 2: Univariate and multivariate COX regression analysis of risk factors for postoperative DFS in OSCC** **younger patients (<60 years)**

|  | **Univariate analysis** | |  | **Multivariate analysis** | |
| --- | --- | --- | --- | --- | --- |
|  | **HR（95%CI）** | ***P*-value** |  | **HR（95%CI）** | ***P*-value** |
| **Gender**  **male vs. female** | **1.619（0.991-2.646）** | **0.055** |  |  |  |
| **Smoking history**  **Smoker vs. non-smoker** | **1.105（0.713-1.713）** | **0.665** |  |  |  |
| **Drinking history**  **Drinker vs. non-drinker** | **1.952（1.258-3.029）** | **0.003** |  | **1.846(1.156-2.48)** | **0.010** |
| **Sites** |  | **0.536** |  |  |  |
| **Tongue** | **Ref.** |  |  |  |  |
| **Gingiva** | **1.167(0.642-2.123)** | **0.612** |  |  |  |
| **Buccal** | **1.624 (0.848-3.109)** | **0.144** |  |  |  |
| **Others** | **0.989 (0.554-1.798)** | **0.970** |  |  |  |
| **Growth pattern** |  | **0.839** |  |  |  |
| **Exogenous** | **Ref.** |  |  |  |  |
| **ulcerative** | **1.022 (0.573-1.823)** | **0.941** |  |  |  |
| **Infiltrating type** | **1.159 (0.657-2.045)** | **0.610** |  |  |  |
| **Clinical stages** |  | **0.008** |  |  | **0.317** |
| **0** | **Ref.** |  |  | **Ref.** |  |
| **I** | **2.063(0.582-7.311)** | **0.262** |  | **2.357(0.662-8.389)** | **0.186** |
| **II** | **1.507(0.436-5.207)** | **0.517** |  | **1.846(0.528-6.455)** | **0.337** |
| **III** | **1.782(0.472-6.723)** | **0.394** |  | **1.663 (0.437-6.324)** | **0.455** |
| **IV** | **3.599(1.115-11.167)** | **0.032** |  | **2.807(0.849-9.282)** | **0.887** |
| **NLR**  **High vs. Low** | **2.201 (1.388-3.491)** | **0.001** |  | **1.085 (0.546-2.157)** | **0.815** |
| **PLR**  **High vs. Low** | **1.297(0.833-2.020)** | **0.249** |  |  |  |
| **LMR**  **High vs. Low** | **0.419 (0.242-0.727)** | **0.002** |  | **1.025（0.444-2.367** | **0.954** |
| **SII**  **High vs. Low** | **1.589 (0.891-2.834)** | **0.117** |  |  |  |
| **PNI**  **High vs. Low** | **0.123 (0.044-0.347)** | **<0.001** |  | **0.407（0.237-0.698）** | **0.001** |
| **ALI**  **High vs. Low** | **0.352 (0.225-0.549)** | **<0.001** |  | **0.571（0.285-1.142）** | **0.133** |
| **SIM**  **High vs. Low** | **2.085 (1.127-3.857)** | **0.019** |  | **0.893（0.287-2.799）** | **0.844** |
| **H-index**  **High vs. Low** | **2.584 (1.541-4.335)** | **<0.001** |  | **1.129（0.489-3.305）** | **0.671** |

**Supplementary Table 3: Univariate and multivariate COX regression analysis of risk factors for postoperative OS in older patients ( ≥60 years)**

|  | **Univariate analysis** | |  | **Multivariate analysis** | |
| --- | --- | --- | --- | --- | --- |
|  | **HR（95%CI）** | ***P*-value** |  | **HR（95%CI）** | ***P*-value** |
| **Gender**  **Male vs. female** | **0.895 (0.570-1.403)** | **0.627** |  |  |  |
| **Smoking history**  **Smoker vs. non-smoker** | **0.953 (0.598-1.519)** | **0.840** |  |  |  |
| **Drinking history**  **Drinker vs. non-drinker** | **0.783 (0.474-1.295)** | **0.341** |  |  |  |
| **Sites** |  | **0.152** |  |  |  |
| **Tongue** | **Ref.** |  |  |  |  |
| **Gingiva** | **1.343 (0.786-2.296)** | **0.281** |  |  |  |
| **Buccal** | **0.979 (0.496-1.933)** | **0.952** |  |  |  |
| **Others** | **0.583 (0.261-1.303)** | **0.188** |  |  |  |
| **Growth pattern** |  | **0.288** |  |  |  |
| **Exogenous** | **Ref.** |  |  |  |  |
| **ulcerative** | **1.427 (0.803-2.536)** | **0.225** |  |  |  |
| **Infiltrating type** | **1.559 (0.867-2.805)** | **0.138** |  |  |  |
| **Clinical stages** |  | **<0.001** |  |  | **<0.001** |
| **0** | **Ref.** |  |  | **Ref.** |  |
| **I** | **3.734 (0.436-31.962)** | **0.229** |  | **3.393 (0.395-29.160)** | **0.266** |
| **II** | **6.143 (0.793-47.592)** | **0.082** |  | **5.673 (0.730-44.072)** | **0.097** |
| **III** | **3.582 (0.373-34.442)** | **0.269** |  | **3.052 (0.316-29.483)** | **0.335** |
| **IV** | **17.578 (2.433-127.003)** | **0.004** |  | **13.410 (1.837-97.916)** | **0.010** |
| **NLR**  **High vs. Low** | **1.940 (1.236-3.044)** | **0.004** |  | **0.644 (0.286-1.446)** | **0.286** |
| **PLR**  **High vs. Low** | **1.972 (1.253-3.105)** | **0.003** |  | **1.312 (0.763-2.256)** | **0.327** |
| **LMR**  **High vs. Low** | **0.451 (0.275-0.740)** | **0.002** |  | **1.604 (0.692-3.715)** | **0.271** |
| **SII**  **High vs. Low** | **2.347 (1.467-3.755)** | **<0.001** |  | **0.945 (0.481-1.854)** | **0.868** |
| **PNI**  **High vs. Low** | **0.744 (0.456-1.314)** | **0.342** |  |  |  |
| **ALI**  **High vs. Low** | **0.434 (0.276-0.694)** | **<0.001** |  | **0.604 (0.290-1.256)** | **0.177** |
| **SIM**  **High vs. Low** | **2.864 (1.731-4.737)** | **<0.001** |  | **1.999 (0.763-5.236)** | **0.158** |
| **H-index**  **High vs. Low** | **2.781 (1.750-4.420)** | **<0.001** |  | **1.448 (0.564-3.718)** | **0.441** |

**Supplementary Table 4: Univariate and multivariate COX regression analysis of risk factors for postoperative DFS in older patients (≥60 years)**

|  | **Univariate analysis** | |  | **Multivariate analysis** | |
| --- | --- | --- | --- | --- | --- |
|  | **HR（95%CI）** | ***P*-value** |  | **HR（95%CI）** | ***P*-value** |
| **Gender**  **Male vs. female** | **0.727 (0.507-1.042)** | **0.083** |  |  |  |
| **Smoking history**  **Smoker vs. non-smoker** | **0.848 (0.582-1.233)** | **0.387** |  |  |  |
| **Drinking history**  **Drinker vs. non-drinker** | **0.704 (0.470-1.056)** | **0.090** |  |  |  |
| **Sites** |  | **0.306** |  |  |  |
| **Tongue** | **Ref.** |  |  |  |  |
| **Gingiva** | **1.414 (0.909-2.199)** | **0.124** |  |  |  |
| **Buccal** | **1.008 (0.580-1.753)** | **0.977** |  |  |  |
| **Others** | **0.946 (0.539-1.658)** | **0.846** |  |  |  |
| **Growth pattern** |  | **0.253** |  |  |  |
| **Exogenous** | **Ref.** |  |  |  |  |
| **ulcerative** | **0.817 (0.522-1.278)** | **0.375** |  |  |  |
| **Infiltrating type** | **1.179 (0.764-1.821)** | **0.456** |  |  |  |
| **Clinical stages** |  | **<0.001** |  |  | **0.006** |
| **0** | **Ref.** |  |  | **Ref.** |  |
| **I** | **1.683 (0.646-4.380)** | **0.286** |  | **0.312 (0.134-0.726)** | **0.007** |
| **II** | **1.736 (0.693-4.439)** | **0.239** |  | **0.514 (0.285-0.925)** | **0.026** |
| **III** | **1.540 (0.534-4.441)** | **0.425** |  | **0.550 (0.324-0.935)** | **0.027** |
| **IV** | **3.796 (1.650-8.733)** | **0.002** |  | **0.471 (0.224-0.990)** | **0.047** |
| **NLR**  **High vs. Low** | **1.651 (1.152-2.366)** | **0.006** |  | **1.057 (0.567-1.969)** | **0.862** |
| **PLR**  **High vs. Low** | **1.783 (1.232-2.525)** | **0.002** |  | **0.763 (0.502-1.161)** | **0.207** |
| **LMR**  **High vs. Low** | **0.683 (0.442-1.005)** | **0.085** |  |  |  |
| **SII**  **High vs. Low** | **1.954 (1.322-2.888)** | **0.001** |  | **0.861 (0.498-1.490)** | **0.594** |
| **PNI**  **High vs. Low** | **0.835 (0.552-1.263)** | **0.393** |  |  |  |
| **ALI**  **High vs. Low** | **0.581 (0.406-0.832)** | **0.003** |  | **1.164 (0.643-2.107)** | **0.617** |
| **SIM**  **High vs. Low** | **1.689 (1.072-2.662)** | **0.024** |  | **1.203 (0.598-2.418)** | **0.604** |
| **H-index**  **High vs. Low** | **1.904 (1.284-2.824)** | **0.001** |  | **1.310 (0.630-2.725)** | **0.470** |

**Supplementary Table 5: Univariate and multivariate COX regression analysis of risk factors for postoperative DFS in male patients**

|  | **Univariate analysis** | |  | **Multivariate analysis** | |
| --- | --- | --- | --- | --- | --- |
|  | **HR（95%CI）** | ***P*-value** |  | **HR（95%CI）** | ***P*-value** |
| **Age**  **<60 years vs. ≥60 years** | **0.844（0.581-1.226）** | **0.374** |  |  |  |
| **Smoking history**  **Smoker vs. non-smoker** | **0.846（0.567-1.263）** | **0.413** |  |  |  |
| **Drinking history**  **Drinker vs. non-drinker** | **1.345（0.909-1.991）** | **0.139** |  |  |  |
| **Sites** |  | **0.607** |  |  |  |
| **Tongue** | **Ref.** |  |  |  |  |
| **Gingiva** | **1.179 (0.717-1.937)** | **0.516** |  |  |  |
| **Buccal** | **1.289 (0.740-2.242)** | **0.370** |  |  |  |
| **Others** | **0.906 (0.555-1.482)** | **0.695** |  |  |  |
| **Growth pattern** |  | **0.808** |  |  |  |
| **Exogenous** | **Ref.** |  |  |  |  |
| **ulcerative** | **1.113 (0.691-1.792)** | **0.661** |  |  |  |
| **Infiltrating type** | **1.173 (0.723-1.903)** | **0.519** |  |  |  |
| **Clinical stages** |  | **0.001** |  |  | **0.089** |
| **0** | **Ref.** |  |  | **Ref.** |  |
| **I** | **2.866 (0.808-10.161)** | **0.103** |  | **2.460 (0.687-8.815)** | **0.167** |
| **II** | **2.131 (0.624-7.274)** | **0227** |  | **2.010 (0.583-6.931)** | **0.269** |
| **III** | **3.411 (0.980-11.873)** | **0.054** |  | **3.011 (0.856-10.594)** | **0.086** |
| **IV** | **4.727 (1.485-15.047)** | **0.009** |  | **3.599 (1.106-11.711)** | **0.033** |
| **NLR**  **High vs. Low** | **1.914 (1.316-2.786)** | **0.001** |  | **1.033 (0.555-1.923)** | **0.918** |
| **PLR**  **High vs. Low** | **1.574 (1.084-2.287)** | **0.017** |  | **0.968 (0.607-1.544)** | **0.891** |
| **LMR**  **High vs. Low** | **0.489 (0.332-0.751)** | **0.001** |  | **1.125 (0.558-2.270)** | **0.742** |
| **SII**  **High vs. Low** | **1.865 (1.225-2.838)** | **0.004** |  | **0.906 (0.480-1.709)** | **0.760** |
| **PNI**  **High vs. Low** | **0.471 (0.303-0.731)** | **0.001** |  | **0.615 (0.377-1.003)** | **0.051** |
| **ALI**  **High vs. Low** | **0.458 (0.315-0.666)** | **<0.001** |  | **0.712 (0.392-1.294)** | **0.265** |
| **SIM**  **High vs. Low** | **2.111 (1.366-3.261)** | **0.001** |  | **1.143 (0.497-2.628)** | **0.753** |
| **H-index**  **High vs. Low** | **2.344 (1.572-3.494)** | **<0.001** |  | **1.464 (0.660-3.248)** | **0.349** |

**Supplementary Table 6: Univariate and multivariate COX regression analysis of risk factors for postoperative OS in female patients**

|  | **Univariate analysis** | |  | **Multivariate analysis** | |
| --- | --- | --- | --- | --- | --- |
|  | **HR（95%CI）** | ***P*-value** |  | **HR（95%CI）** | ***P*-value** |
| **Age**  **<60 years vs.**  **≥60 years** | **3.719 (1.574-8.787)** | **0.003** |  | **2.660(1.073-6.592)** | **0.035** |
| **Smoking history**  **Smoker vs. non-smoker** | **1.547 (0.479-4.992)** | **0.466** |  |  |  |
| **Drinking history**  **Drinker vs. non-drinker** | **0.047 (0.001-90.400)** | **0.429** |  |  |  |
| **Sites** |  | **0.124** |  |  | **0.497** |
| **Tongue** | **Ref.** |  |  | **Ref.** |  |
| **Gingiva** | **2.144 (1.105-4.159)** | **0.024** |  | **0.875 (0.424-1.809)** | **0.719** |
| **Buccal** | **1.410 (0.575-3.459)** | **0.453** |  | **0.611 (0.236-1.579)** | **0.309** |
| **Others** | **0.869 (0.199-3.802)** | **0.852** |  | **0.375 (0.082-1.710)** | **0.205** |
| **Growth pattern** |  | **0.780** |  |  |  |
| **Exogenous** | **Ref.** |  |  |  |  |
| **ulcerative** | **0.837 (0.404-1.738)** | **0.633** |  |  |  |
| **Infiltrating type** | **1.079 (0.533-2.182)** | **0.833** |  |  |  |
| **Clinical stages** |  | **<0.001** |  |  | **<0.001** |
| **0** | **Ref.** |  |  | **Ref.** |  |
| **I** | **1.575 (0.164-15.144)** | **0.694** |  | **1.399 (0.144-13.556)** | **0.772** |
| **II** | **2.290 (0.267-19.606)** | **0.449** |  | **2.306 (0.267-19.888)** | **0.447** |
| **III** | **0.001 (<0.001-0.005)** | **0.978** |  | **0.001 (<0.001-0.005)** | **0.979** |
| **IV** | **13.754 (1.885-100.361)** | **0.010** |  | **12.749 (1.713-94.878)** | **0.013** |
| **NLR**  **High vs. Low** | **1.840 (0.999-3.390)** | **0.050** |  | **0.459 (0.165-1.276)** | **0.135** |
| **PLR**  **High vs. Low** | **1.553 (0.866-2.787)** | **0.140** |  |  |  |
| **LMR**  **High vs. Low** | **0.584 (0.230-1.480)** | **0.257** |  |  |  |
| **SII**  **High vs. Low** | **2.171 (1.121-4.204)** | **0.022** |  | **0.812 (0.357-1.850)** | **0.620** |
| **PNI**  **High vs. Low** | **0.736 (0.380-1.425)** | **0.364** |  |  |  |
| **ALI**  **High vs. Low** | **0.409 (0.226-0.739)** | **0.003** |  | **0.546 (0.190-1.572)** | **0.262** |
| **SIM**  **High vs. Low** | **3.057 (1.363-6.856)** | **0.007** |  | **1.973 (0.607-6.412)** | **0.258** |
| **H-index**  **High vs. Low** | **3.036 (1.592-5.788)** | **0.001** |  | **1.096 (0.326-3.682)** | **0.882** |

**Supplementary Table 7: Univariate and multivariate COX regression analysis of risk factors for postoperative DFS in female patients**

|  | **Univariate analysis** | |  | **Multivariate analysis** | |
| --- | --- | --- | --- | --- | --- |
|  | **HR（95%CI）** | ***P*-value** |  | **HR（95%CI）** | ***P*-value** |
| **Age**  **<60 years vs.**  **≥60 years** | **1.780 (1.098-2.866)** | **0.019** |  | **0.744(0.436-1.269)** | **0.278** |
| **Smoking history**  **Smoker vs. non-smoker** | **1.345 (0.545-3.318)** | **0.521** |  |  |  |
| **Drinking history**  **Drinker vs. non-drinker** | **0.047 (0.001-7.784)** | **0.241** |  |  |  |
| **Sites** |  | **0.233** |  |  | **0.804** |
| **Tongue** | **Ref.** |  |  | **Ref.** |  |
| **Gingiva** | **1.639 (1.022-2.629)** | **0.040** |  | **0.959 (0.559-1.643)** | **0.878** |
| **Buccal** | **1.129 (0.596-2.140)** | **0.710** |  | **0.728 (0.370-1.432)** | **0.358** |
| **Others** | **1.224 (0.541-2.916)** | **0.648** |  | **0.835 (0.342-2.038)** | **0.691** |
| **Growth pattern** |  | **0.085** |  |  |  |
| **Exogenous** | **Ref.** |  |  |  |  |
| **ulcerative** | **0.629 (0.365-1.083)** | **0.095** |  |  |  |
| **Infiltrating type** | **1.126 (0.691-1.834)** | **0.634** |  |  |  |
| **Clinical stages** |  | **<0.001** |  |  | **0.002** |
| **0** | **Ref.** |  |  | **Ref.** |  |
| **I** | **1.287 (0.494-3.349)** | **0.606** |  | **1.240 (0.474-3.245)** | **0.662** |
| **II** | **1.265 (0.495-3.233)** | **0.624** |  | **1.312 (0.508-3.386)** | **0.575** |
| **III** | **0.408 (0.082-2.021)** | **0.272** |  | **0.411 (0.083-2.047)** | **0.278** |
| **IV** | **3.473 (1.488-8.109)** | **0.004** |  | **2.974 (1.235-7.160)** | **0.015** |
| **NLR**  **High vs. Low** | **1.828 (1.176-2.842)** | **0.007** |  | **1.128 (0.558-2.280)** | **0.737** |
| **PLR**  **High vs. Low** | **1.608 (1.059-2.433)** | **0.026** |  | **0.802 (0.506-1.273)** | **0.350** |
| **LMR**  **High vs. Low** | **0.736 (0.355-1.523)** | **0.408** |  |  |  |
| **SII**  **High vs. Low** | **1.931 (1.171-3.183)** | **0.010** |  | **0.877 (0.459-1.677)** | **0.691** |
| **PNI**  **High vs. Low** | **0.720 (0.448-1.159)** | **0.176** |  |  |  |
| **ALI**  **High vs. Low** | **0.490 (0.317-0.756)** | **0.001** |  | **1.699 (0.829-3.481)** | **0.148** |
| **SIM**  **High vs. Low** | **1.348 (0.621-2.923)** | **0.450** |  |  |  |
| **H-index**  **High vs. Low** | **1.897 (1.116-3.226)** | **0.018** |  | **1.287 (0.578-2.867)** | **0.536** |
