# Supplementary figures and images for: The comparison of the prognostic value of different inflammation-related indicators in patients with oral squamous cell carcinoma
Source: Front Genet. 2025 Aug 18;16:1652603. doi: 10.3389/fgene.2025.1652603 (PMC12399640; doi:10.3389/fgene.2025.1652603)

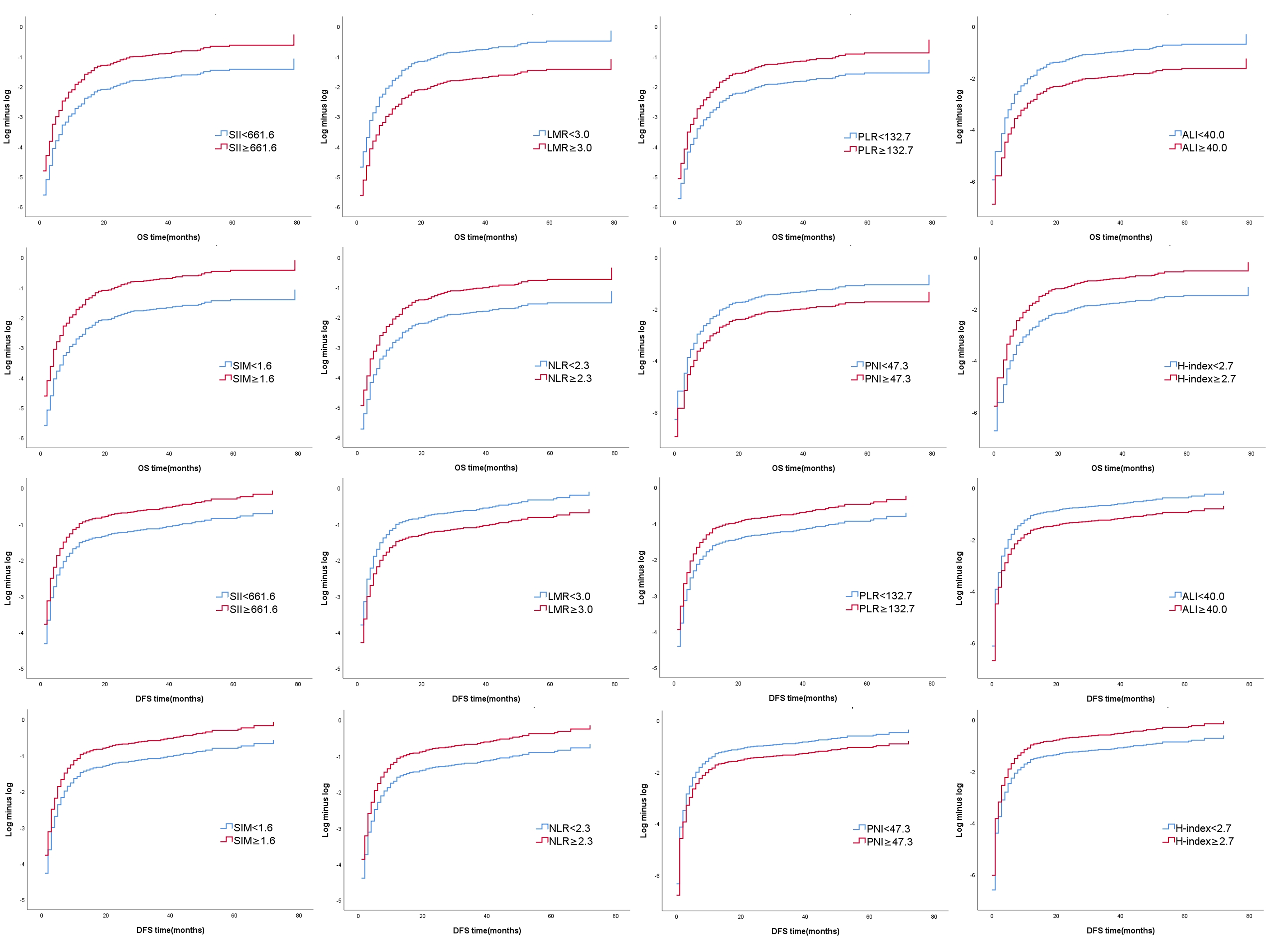

Supplement: Supplementary file 2 [file Image1.tif]
